# Climate change and spatio-temporal trend analysis of climate extremes in the homogeneous climatic zones of Pakistan during 1962-2019

**DOI:** 10.1371/journal.pone.0271626

**Published:** 2022-07-27

**Authors:** Firdos Khan, Shaukat Ali, Christoph Mayer, Hamd Ullah, Sher Muhammad

**Affiliations:** 1 School of Natural Sciences (SNS), National University of Sciences and Technology (NUST), Islamabad, Pakistan; 2 Global Change Impact Studies Centre (GCISC), Ministry of Climate Change, Islamabad, Pakistan; 3 Geodesy and Glaciology (KEG), Bavarian Academy of Sciences and Humanities, Munich, Germany; 4 Department of Mathematics and Statistics, International Islamic University, Islamabad, Pakistan; 5 International Centre for Integrated Mountains Development, Kathmandu, Nepal; Universiti Teknologi Malaysia, MALAYSIA

## Abstract

Climate extremes, such as heat waves, droughts, extreme rainfall can lead to harvest failures, flooding and consequently threaten the food security worldwide. Improving our understanding about climate extremes can mitigate the worst impacts of climate change and extremes. The objective here is to investigate the changes in climate and climate extremes by considering two time slices (i.e., 1962–1990 and 1991–2019) in all climate zones of Pakistan by utilizing observed data from 54 meteorological stations. Different statistical methods and techniques were applied on observed station data to assess changes in temperature, precipitation and spatio-temporal trends of climatic extremes over Pakistan from 1962 to 2019. The Mann-Kendal test demonstrated increasing precipitation (DJF) and decreasing maximum and minimum temperatures (JJA) at the meteorological stations located in the Karakoram region during 1962–1990. The decadal analysis, on the other hand, showed a decrease in precipitation during 1991–2019 and an increase in temperature (maximum and minimum) during 2010–2019, which is consistent with the recently observed slight mass loss of glaciers related to the Karakoram Anomaly. These changes are highly significant at 5% level of significance at most of the stations. In case of temperature extremes, summer days (SU25) increased except in zone 4, TX10p (cold days) decreased across the country during 1962–1990, except for zones 1 and 2. TX90p (warm days) increased between 1991–2019, with the exception of zone 5, and decreased during 1962–1990, with the exception of zones 2 and 5. The spatio-temporal trend of consecutive dry days (CDD) indicated a rising tendency from 1991 to 2019, with the exception of zone 4, which showed a decreasing trend. PRCPTOT (annual total wet-day precipitation), R10 (number of heavy precipitation days), R20 (number of very heavy precipitation days), and R25mm (very heavy precipitation days) increased (decreased) considerably in the North Pakistan during 1962–1990 (1991–2019). The findings of this study can help to address some of the sustainable development goals related climate action, hunger and environment. In addition, the findings can help in developing sustainable adaptation and mitigation strategies against climate change and extremes. As the climate and extremes conditions are not the uniform in all climate zone, therefore, it is suggested to the formers and agriculture department to harvest crops resilient to the climatic condition of each zone. Temperature has increasing trend in the northern Pakistan, therefore, the concerned stakeholders need to make rational plans for higher river flow/flood situation due to snow and glacier melt.

## 1. Introduction

Climate change has been attributed to human-caused activities, especially increased greenhouse gases emissions after the mid-twentieth century [1–4]. Climate change is causing a number of threats, including an increase in the frequency of extreme events and other disasters [5,6]. Evidence of observed changes in extremes such as heavy precipitation, heatwaves, tropical cyclones, and droughts, and, in particular, the attribution of these extremes to human influence, has strengthened since AR5 (Fifth Assessment Report) [6]. The societal infrastructure is becoming more vulnerable to climate extremes, which are exacerbated by climate change [7–9]. Climate extremes such as droughts, floods, heatwaves, and typhoons have geographically different economic, health, social, ecological, and environmental impacts [10–15].

Many approaches have been developed to explore variability and trends of climate extremes using observed precipitation and temperature data around the world. For example, Grima et al. [16] investigated the variability of temperature and precipitation in the Upper Huai River Basin of China using yearly time series data from 1960 to 2016, concluded a decreasing trend in precipitation and a considerably increasing trend in temperature at all meteorological stations. Bougara et al. [17] investigated the variability of rainfall in the Tafna basin in northeastern Algeria using observed data from 1979 to 2011, resulted in heterogeneous results in different stations. Asfaw et al. [18] performed variability and trend analyses of temperature using data from 1901 to 2014 in the Woleka sub-basin in northcentral Ethiopia and found a significant increasing trend in mean and minimum average temperatures. Other studies that are based on observe station data on variability and trend analysis of temperature and precipitation in various regions of the globe include: [19–24].

Most of the studies conducted in Pakistan considered a sub-region of Pakistan for their analyses [25–29]. For instance, Alam et al. [25] considered three meteorological stations in the north-western part (Khyber Pakhtoonkhwa province) and used Mann Kendall (MK) test for trend analysis using daily observed data for the duration of 1960–2020. Safdar et al. [27] investigated the climate change indicators and spatio-temporal shift in Monsoon pattern by using observed data for the duration of 1980–2017. They concluded a warming tendency and a decrease in the rainfall in the Monsoon region of Pakistan. Hussain et al. [26] investigated variability and trends in precipitation and temperature over the northern part of Pakistan by using observed meteorological data (duration of 1955–2016 for some station and 1995–2012 for other stations). They concluded elevation-dependent conclusions in the target location, for instance, they noted increasing trend in maximum temperature and precipitation at station with elevation 1500–2800 meters in contrast to elevation less than 1500 meters during 1955–2016. Further studies which investigated variability and trends analysis in the sub-regions of the country include [28–30].

Studies related to precipitation and temperatures’ extremes in different parts of the world revealed that these extremes are increased in terms of frequency, intensity and persistence [31–33]. The study of Sun et al. [33] concluded that the extreme precipitation has increased at two-third stations globally during 1950–2018. Frich et al. [34] investigated about the possible changes in climate extreme over the whole glob using observed data for the duration of 1946–1999. They noted a significant increase and a decrease in the number of warm summer nights and the number of frost days, respectively, and a significant increase in heavy precipitation events.

Sheikh et al. [35] derived climate extremes for temperature (for the duration of 1971–2000) and precipitation (for the duration of 1961–2000) using observed data over Pakistan, India, Bangladesh, Nepal and Sri Lanka. Their results indicate an increase in precipitation extremes in South Asia, consistent with the global average. Rizvi et al. [36] investigated temperature and precipitation extreme at four meteorological stations (Islamabad, Muree, Jhelum and Mianwali) using observed data for the duration of 1960–2013. Their findings concluded that most of the temperature extremes have positive trend except FDO at Islamabad and Jhelum stations.

Glaciers play significant role in terms of water availability in the mountainous regions in the world [37,38]. Most of the mountain glaciers worldwide are losing mass [39,40]. In contrast, Glaciers in the Karakoram and adjacent Kunlun Shan seem to be stable or even growing in the recent past [41–43]. The glaciers stability is consistent with temperature trends in the Upper Indus Basin (UIB) between 1961 and 2000, investigated by Fowler et al. [44], indicating decreasing summer temperatures and runoff. These studies used somehow outdated climate data till 2000 and call for updated investigation. With the recently recorded climate data, it can update the recent trends and potentially explain the Karakoram anomaly. In addition, spatio-temporal trend analysis of precipitation and temperature extremes can provide information about the frequency, intencity and duration of extremes events like floods, heatwaves and droughts etc. Towards this end, a special section is presented to investigate climate change and significance of the temperature and precipitation extremes at each meteorological station in the northern part of Pakistan.

Various studies conducted recently explored a relationship between heatwaves, high-temperature extremes, and population exposure during the 21^st^ Century. For instance, Iyakaremye et al. [45] concluded that by the mid of 21^st^ century, the population exposure is expected to increase by ~ 28% (25%) in comparison to the baseline duration under SSP5-8.5 (SSP2-4.5) in Africa. May parts of Africa has experienced intensified and more frequent hot days and nights recently which clearly indicating a shift in the African climate [46]. Projected results about precipitation suggested uncertainties over Pakistan [47,48], however, some studies suggested that precipitation has mixed trend (decreasing trend for some time periods and increasing trend for other time periods) over the northern Pakistan in the future. This demonstrate that it is imperative to understand better the condition of climate change and extremes, particularly in the UIB as it will have impacts on water availability, hydropower generation, agriculture and the livelihood of inhabitants downstream. In addition, this can assist policy makers in terms of planning for better mitigation and adaptation measures in the future.

A different approach is adapted in contrast to the previous studies to investigate climate change signals and climate extremes’ analysis over Pakistan for the duration of 1962–2019 (making two time slices, i.e., 1962–1990 and 1991–2019). Initially, the change in the mean values for precipitation and temperature between two-time slices is tested for possible significance separately. The spatio-temporal trend analysis of climate extremes is then performed zone wise for each time duration. A comparison is made between the two time periods to see the possible changes in climate change and climate extremes. The assessment is drawn by using various statistical techniques, e.g., probability density functions, spatio-temporal trend analysis and testing the significance of climate extremes.

The major aims of this study are: (1) to test the significance of changing climate in two-time durations for each climate zone in Pakistan and each station in the HKH (Hindukush-Karakoram-Himalaya) region; (2) spatio-temporal trend analysis of climate extremes in each climate zone and for each station in the HKH region. Moreover, the results of this study may help to address some of the Sustainable Development Goals (SDGs) of the United Nation Development Programme (UNDP) related to climate action, water, hunger and environment. The remaining paper is structured as: section 2 is for study area and data; section 3 presents the methodology; section 4 consists of results; section 5 is reserved for discussion and section 6 presents conclusions about this study.

## 2. Study area and data

**Fig 1:**
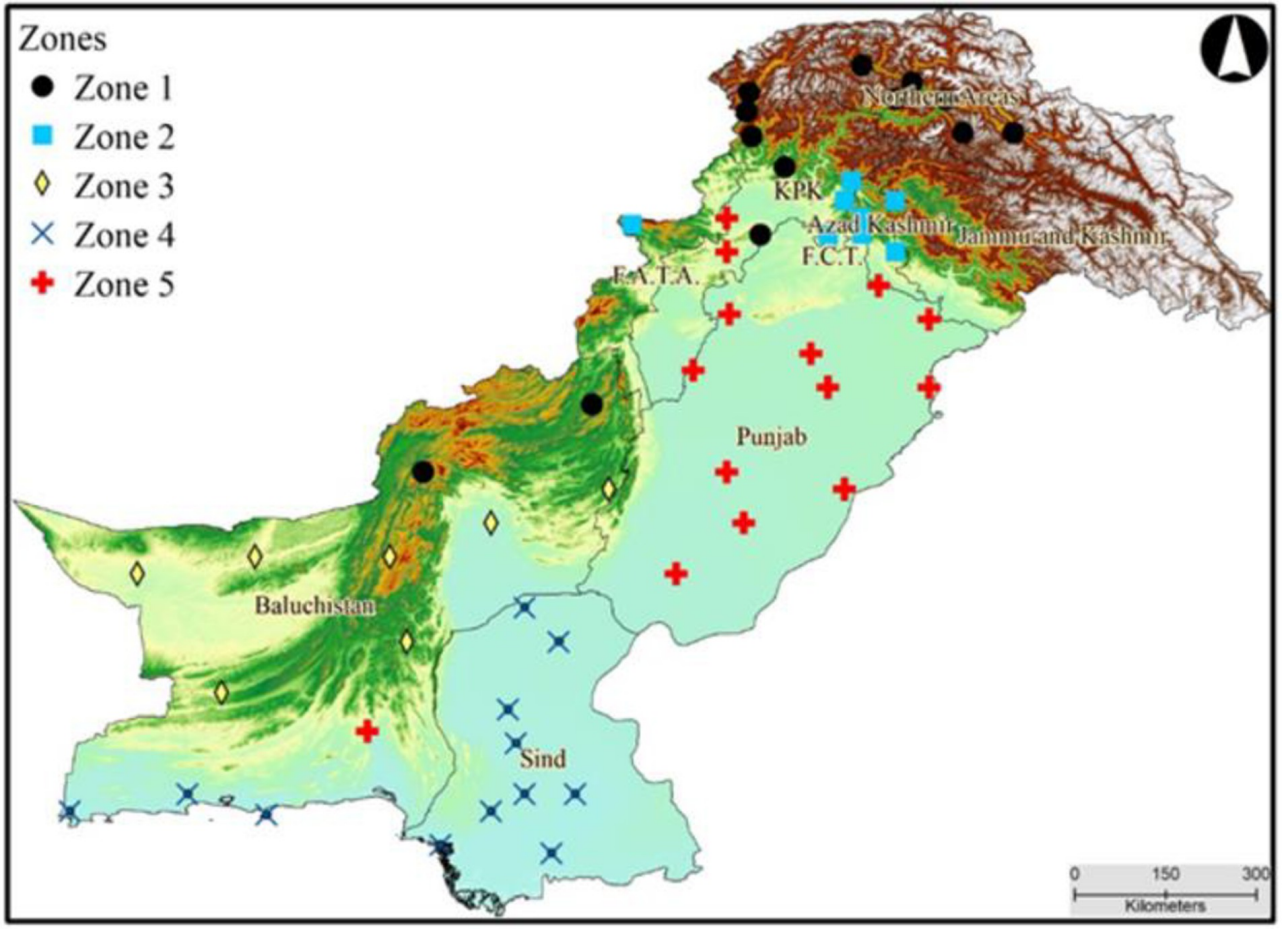
Five homogeneous climate zones of Pakistan. We used open access SRTM data to generate Fig 1, there is no copyright restrictions to use the data. *Source*: *NASA Shuttle Radar Topography Mission (SRTM) (2013)*. *Shuttle Radar Topography Mission (SRTM) Global*. *Distributed by OpenTopography*. *https://doi.org/10.5069/G9445JDF*. *Accessed*: *2022-04-09*.

The target location of this study is Pakistan which has 881,913 square kilometers area and approximately 222 million inhabitants [49–51]. Pakistan lies in the northern hemisphere with latitude of 23.4–37.3N and longitude of 60.5–78.5E. It has a diversity in climate as it has Arabian Sea in the south and the world’s highest mountains (Himalaya, Hindukush and Karakoram) in the north. The country has four climate seasons (Winter = December-February (DJF), Spring = March-May (MAM), Summer = June-August (JJA), Autumn = September-November (SON)) and consequently significant spatial and temporal variation in the climate. In Fig 1, zone 1 comprises cold regions include northern and central-western parts of the country. Zone 2 comprised of Islamabad Capital Territory (ICT), some parts of Azad-Jammu and Kashmir and Khyber Pakhtoonkhwa which is part of the monsoon dominated region and receive maximum rainfall during the monsoon season. Zone 3 is the south-western part of the country and has least rainfall in a year as compared to the other regions of the country. Zone 4 includes the south-eastern and coastal areas in the south of the country. Zone 5 is the central-eastern part of the country and due to its fertile land, it is considering the food basket of the country. Observed daily climate data (maximum, minimum temperature and precipitation) for the period of 1962–2019 was acquired from Pakistan Meteorological Department (PMD) for fifty-four (54) meteorological stations across the country. The spatial distribution of meteorological stations is shown in Fig 1. The data was divided into two independent climatic periods (1962–1990 and 1991–2019) for the assessment of climate change and extremes analysis over Pakistan.

## 3. Methodology

### 3.1. Inconsistencies in the data

There were some missing values in the maximum, minimum temperature and precipitation particularly at the stations situated at high elevation. There are various statistical methods available to address the issue of missing values, for instance, multivariate regression, interpolation, imputation etc. In this study, imputation method (a regression-based approach) is used at multiple stations to preserve the spatial dependence structure. Multiple chains have been run and the convergence is assessed in each chain after a specified number of iterations. The number of iterations depend on researcher and the nature of the study [52]. However, the convergence of the process can be assessed at each iteration. The process can be stopped if there are no significant changes during the successive iterations. It is important to note that the inhomogeneous data need to be homogenized before the start of further analysis [53]. Inhomogeneities in data may occur due to breaks, changes or disorder in measuring instruments, observing practices of enumerators, location, and environment of the stations. The standard normal homogeneity tests (SNHT) are useful tools to assess the homogeneity, consistency and to detect the spikes and outliers in daily time series [54–57]. The commonly used SNHT for testing the homogeneity of data include Buishand Range test, Pettitt test, and Von Neumann Ratio test. In this study we have used Buishand Range test for testing the homogeneity of the data which can identify the month/year where the gap occurs. For further details about the above-mentioned homogeneity tests, we refer to [58].

### 3.2. Climate change assessment

For climate change assessment, the average differences between the two time slices are tested for possible statistical significance at a 5% level of significance. This is carried out under the hypothesis testing procedures of the student t-test. Under this procedures, the null hypothesis states that there is no difference between the average climatology of two-time slices where the alternative hypothesis states opposite to the null hypothesis. The hypothesis under the student t-test can be formulated as:

$$H_{0}:\;on\;the\;average\;there\;is\;no\;change\;in\;the\;climatology\;of\;both\;time\;slices$$

Vs

$$H_{A}:\;on\;the\;average\;there\;is\;change\;in\;the\;climatology\;of\;both\;time\;slices$$

Alternatively, it can be written as:

$$H_{o}:\;\mu_{1991-2019}-\;\mu_{1962-1990}\;=0$$

Vs

$$H_{A}:\;\mu_{1991-2019}-\;\mu_{1962-1990}\;\neq 0$$

Where *H*_0_ and *H*_*A*_ represent null and alternative hypothesis, respectively. The notation *μ*_1962–1990_ and *μ*_1991–2019_ represent the average climatology during 1962–1990 and 1991–2019, respectively. Here the average climatology means average temperature or precipitation for the mentioned time duration. The change between the two time slices is significant at 5% level of significance if the p-value of the test is less than or equal to 0.05. To assess the detailed behaviors of zone-wise climatology, this analysis was carried out on a seasonal as well as on annual basis.

### 3.3. Trend analysis

For trend analysis, a non-parametric test, the modified version of MK (mMK) test has been used initially proposed by [59] and widely uses in hydrology and climatology for trend analysis (e.g., [60,61]). The null hypothesis in MK test is “no monotonic trend is present in the data”. Which means that if the p-value of the test is less than or equal to 0.05, then it is concluded that there is a trend in the data at 5% level of significance. For further details about the test statistic and calculation of the MK test, we refer to [62].

### 3.4. Climate extremes analysis

Climate extremes related to temperature and precipitation were calculated for both time slices. To assess the changes in climate extremes, probability density functions were drawn for both time slices and a comparison is made between them. To investigate spatial variability and changes, the changes in climate extremes were interpolated to the target location by using a geostatistical interpolation method called Kriging. The analysis provides information about the changes in climate extremes during the two-time slices over the full domain. Finally, the statistical significance of the climate extremes was performed by using a 5% level of significance. A comparison was made between climate extremes in both time slices for temperature and precipitation in each climate zone in terms of statistical significance. For definition and unit of measurement of climate extremes, we refer to [63,64]. A summary of the climate extremes used in this study is given in Annex-01.

### 3.5. Climate change assessment and extremes’ analysis in HKH region

HKH comprises one of the world’s largest non-polar glaciers [65–67], therefore, climate change and extremes can have significant impacts on snow and glacier melt which can further alter the hydrological regime of this region. Different studies conducted about trends in temperature, precipitation and extremes over Pakistan recently. For instance, the results of Bhatti et al. [68] suggested that the linear trend of all precipitation extreme indices showed an increasing tendency over Pakistan during 1980–2016. The study of Ullah et al. [69] illustrated that the spatial distributions of Tmin and Tmax showed a warming trend over the whole country, however, the seasonal and annual Tmin (Tmax) exhibited sharp increasing trends in the southern, southwestern and southeastern (northern and southwestern mountainous) regions of Pakistan. Elevation dependent significant fluctuations have been noted in long-term precipitation trend (during 1980–2016) in Pakistan with these categories: decreases in 500–1000m and 1000–1500m elevation zones; significant increasing trend in elevation zones of ≤500m and≥1500 m [70]. Strong variability has been noted in precipitation during 1986–2005 over Pakistan, particularly in the northern parts of the country. For instance, a positive trend in the precipitation variability was noted during the winter season in Himalaya and Karakorum region while a negative trend was noted during pre-monsoon season in the Karakorum and Himalayas regions [71]. Studies related to extremes analysis suggested that the precipitation and temperature extremes like warm nights, summer days, warm days, warmest day, and coldest day increased during 1980–2016 over Pakistan [72,73]. Archer [74] investigated that with the rise of 1°C in average temperature can cause an increase of 16–17% in river flow in the upper Indus Basin.

Few previous studies compared temperature and precipitation trends on local scale glaciers in the Karakoram and Himalayas in the UIB [75,76]. These findings suggest that slight temperature and precipitation supported the marginal retreating trends. The glaciers changes in correlation with the local climate improves the assessment of contemporary and future glaciers behavior under the changing climate. Our long-term climate assessment would improve the cryosphere changes in the glacier’s rich mountainous region in the context of the Karakoram Anomaly.

**Fig 2:**
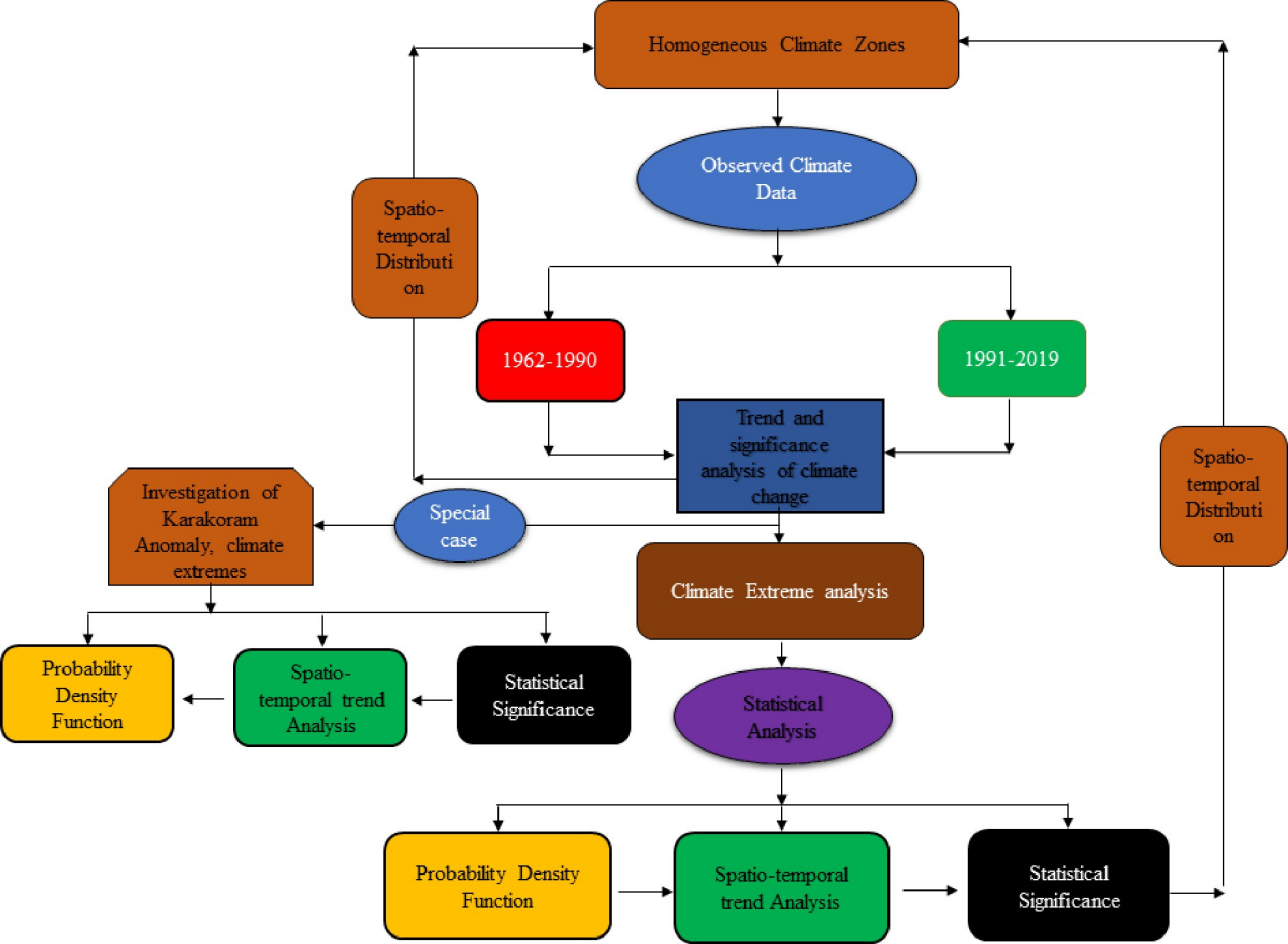
Schematic presentation of the methodology of the study.

There are seven meteorological stations with long-term observations in this region as shown in Table 1. The long-term meteorological changes were investigated by using observed data at each station. The significance of difference (average changes) between the two periods (1962–1990 and 1991–2019) was tested by utilizing a t-test to see whether it is significantly changing or not for each station separately on a seasonal basis. This can help to see the significance of the changes in average temperature and precipitation on a seasonal basis in different parts of the Karakoram region. In addition, the mMK test was used to identify a monotonic trend in temperature and precipitation. Finally, climate extremes were calculated for each station to see whether it is behaving differently than the zone wise climate extremes. This special part may disclose the latest status of climate change in different parts of the Karakoram and may update the present status of Karakoram anomaly which was first investigated by [43]. The details of meteorological station in Northern Pakistan are given in Table 1, with four stations are in the Karakoram and the remaining in the Himalaya and Hindukush region. The step-by-step methodology of this study is presented in Fig 2.

**Table 1:** Brief information about meteorological stations situated in Northern Pakistan and used for Karakoram Anomalies investigation.

S. No	Station	Latitude	Longitude	Mountain range	Altitude
1	Astore	35 20	74 54	Himalaya	2,168.0 meters
2	Bunji	35.75	74.75	**Karakoram**	1,372.0 meters
3	Chilas	35.75	74.00	Hindukush	1,250.0 meters
4	Darosh	35.50	71.75	Hindukush	1,464.0 meters
5	Gilgit	36.00	74.50	**Karakoram**	1,460.0 meters
6	Gupis	36.25	73.50	**Karakoram**	2,156.0 meters
7	Skardu	35.25	75.75	**Karakoram**	2,317.0 meters

## 4. Results

Before the start of formal analysis, the data was investigated carefully for missing values and inhomogeneity. After filling the missing values using imputation, the results of SNHT test show that the data is homogeneous in time. Therefore, there was no need of any adjustment. The climate change extreme results of this study are discussed zone-wise and thereafter the new findings about the Karakoram anomaly. Considering the importance of the Karakoram region, climate extremes analysis is also presented for each station separately in this region.

### 4.1. Estimation

#### Zone 1

The difference between the two-time slices (1991–2019 and 1962–1990) was tested for possible significance in temperature and precipitation on a seasonal basis. A significant increase in temperature during DJF and MAM and a significant decrease during JJA and SON was observed as shown in Table 2. Precipitation increased significantly during all seasons except JJA. The mMK tests’ results for maximum, minimum temperature and precipitation are shown in Table 3. For zone 1, the mMK test for precipitation’s change shows significantly decreasing trend with value -0.0618. The values of mMK statistic for changes in maximum and minimum temperature are 0.0752 and 0.0762, respectively, indicating increasing trends. The mMK test’s results support the Karakoram anomaly during the 1962–1990 given in Table 5, however, the decadal analysis during the 1990–2019 offers complex results (discussed in detail under the Climate Change assessment and extremes’ analysis in HKH region). During the last decade, the trend analysis show that precipitation and temperature have decreasing and increasing trends, respectively, at the stations located in the Karakoram region.

**Table 2:** Test of significance of the difference between 1991–2019 and 1962–1990 about average temperature and precipitation for all seasons and climate zones.

Variable	Season	Zone-1	Zone-2	Zone-3	Zone-4	Zone-5
Average Temperature	DJF	1.8174***	0.0358	0.3453***	0.6908***	0.5422***
	MAM	1.2477***	0.3193***	0.1619**	0.3604***	0.9524***
	JJA	-1.2613***	0.0654**	-0.4206***	-0.1703***	0.0501
	SON	-0.5780***	-0.0100	0.3273***	0.3578***	0.4525***
	**Overall**	0.3016**	**0.1033**	**0.1017**	0.3077***	0.4992***
Precipitation	DJF	2.4593***	4.8609***	0.1886	0.2085	2.2729****
	MAM	0.7455**	2.0928**	0.7138***	0.1233	0.7285
	JJA	0.0995	5.5510***	1.9361***	1.1500**	4.9269***
	SON	0.9809***	3.4153***	0.5277***	1.5977***	3.7380***
	**Overall**	1.0649**	3.9773***	0.8455***	0.7703**	2.9174***

Note: Where “***” and “**” indicate significance of the test at 1% and 5% level of significance. DJF = December, January, February; MAM = March, April, May; JJA = June, July, August; SON = September, October, November.

**Table 3:** Man-Kendall test for testing trend in each duration (1962–1990, 1991–2019, Change = difference between 1991–2019 and 1962–1990). Each series has two columns, first is for the estimated value of Kendall *τ* and second is for their corresponding p-value.

S. No	Zones	Precipitation						Max. TMP						Min. TMP					
		1962–1990		1991–2019		Changes (1991-2019-1962-1990)		1962–1990		1991–2019		Changes (1991-2019-1962-1990)		1962–1990		1991–2019		Changes (1991-2019-1962-1990)	
		Kendall *τ*	P-Value	Kendall *τ*	P-Value	Kendall *τ*	P-Value	Kendall *τ*	P-Value	Kendall *τ*	P-Value	Kendall *τ*	P-Value	Kendall *τ*	P-Value	Kendall *τ*	P-Value	Kendall *τ*	P-Value
1	Z-1	0.0453698	5.476e-11	-4.554031e-02	3.078e-11	-6.181994e-02	2.2e-16	-2.941424e-03	0.6556	2.879244e-02	1.266e-05	7.515349e-02	2.2e-16	-1.370601e-02	0.03766	1.215344e-02	0.06534	7.616308e-02	2.2e-16
2	Z-2	-8.099797e-03	0.2362	-1.955711e-02	0.004891	-1.409285e-02	0.03446	3.382319e-03	0.608	2.200206e-02	0.00084	2.933388e-02	8.644e-06	-1.510223e-02	0.02201	2.225517e-02	0.00074	9.385996e-02	< 2.2e-16
3	Z-3	1.265816e-01	< 2.2e-16	-1.866923e-02	0.01437	-8.902188e-02	< 2.2e-16	-1.167005e-02	0.07684	2.368513e-02	0.0003292	6.451906e-02	< 2.2e-16	2.921715e-02	9.437e-06	1.949849e-03	0.7675	-6.366869e-02	< 2.2e-16
4	Z-4	2.655612e-02	0.0005049	7.844310e-03	0.3084	-9.856097e-03	0.1802	1.122931e-02	0.08862	1.109748e-02	0.09244	-2.111771e-02	0.001362	-4.265234e-03	0.5178	6.707298e-02	2.2e-16	1.583697e-01	< 2.2e-16
5	Z-5	1.998956e-02	0.005168	-2.121013e-02	0.002958	-2.239363e-02	0.001017	1.140568e-02	0.08369	9.684728e-03	0.1419	-1.569585e-02	0.01729	7.689117e-03	0.2436	2.872170e-02	1.328e-05	5.604884e-02	< 2.2e-16

**Fig 3:**
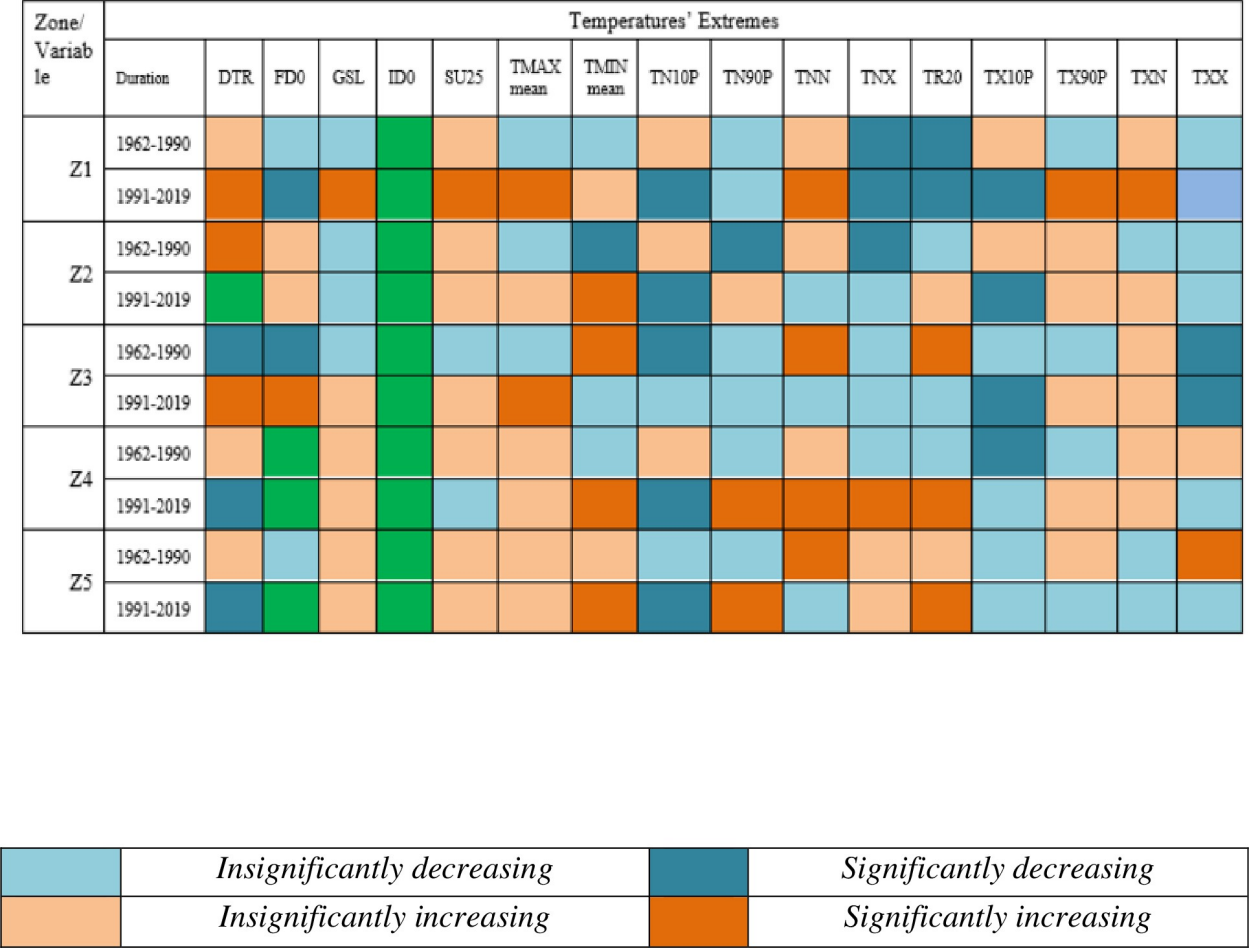
Climate extremes related to temperature for each climatic zone and two independent time duration. Each color has specific meaning and given in note mention with Fig 4. *Note*: *The colors represent different status for each extreme mentioned in these figures*. *Green color shows no changes*.

**Fig 4:**
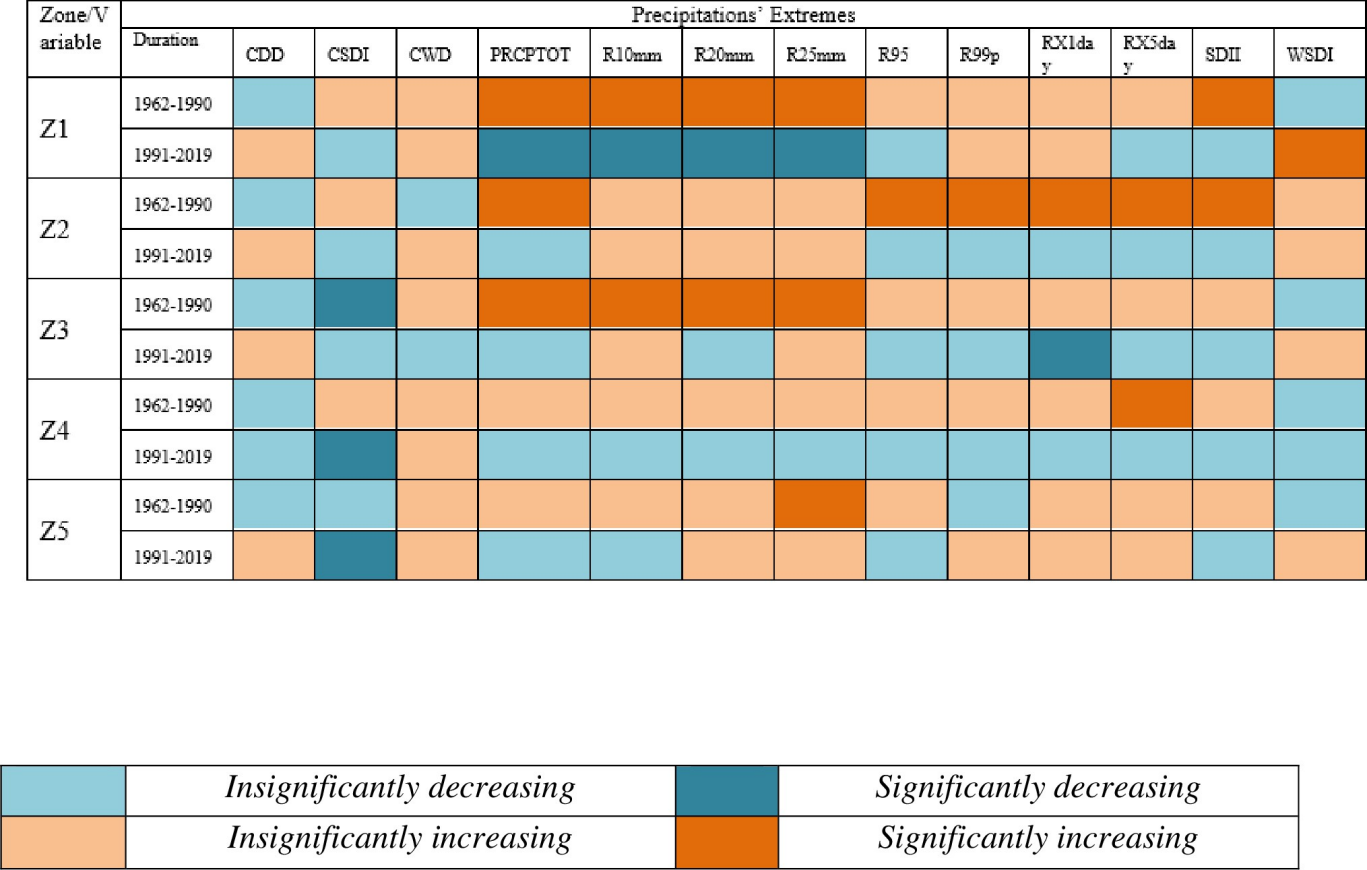
Climate extremes related to precipitation for each climatic zone and two independent time duration. Each color has specific meaning and given below. *Note*: *The colors represent different status for each extreme mentioned in these figures*. *Green color shows no changes*.

**Fig 5:**
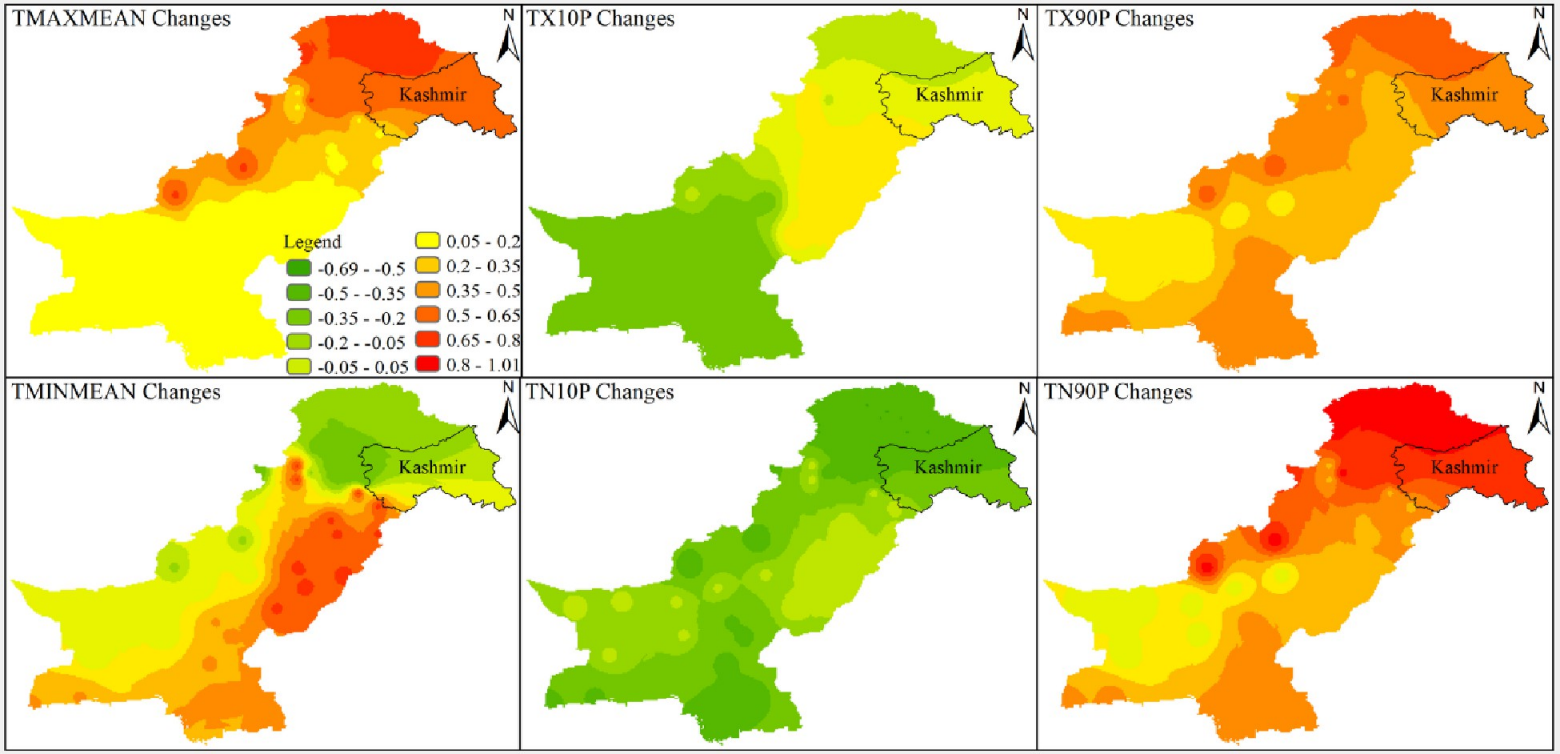
Spatial distribution of changes during 1991–2019 with respect to 1962–1990 of different climate extremes across Pakistan. The extremes are TMAXMean, TX10P, TX90P, TMINMean, TN10P, and TN90P. The legend used in top right panel is applicable to all parts of this figure. The shapefile used for this figure is available at https://data.humdata.org/dataset/pakistan-union-council-boundaries-along-with-other-admin-boundaries-dataset.

**Fig 6:**
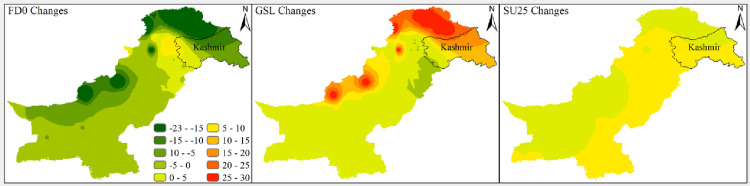
Spatial distribution of changes during 1991–2019 with respect to 1962–1990 of different climate extremes across Pakistan. The extremes are FD0, GSL, and SU25. The legend used in the first panel is applicable to all parts of this figure. The shapefile used for this figure is available at https://data.humdata.org/dataset/pakistan-union-council-boundaries-along-with-other-admin-boundaries-dataset.

**Fig 7:**
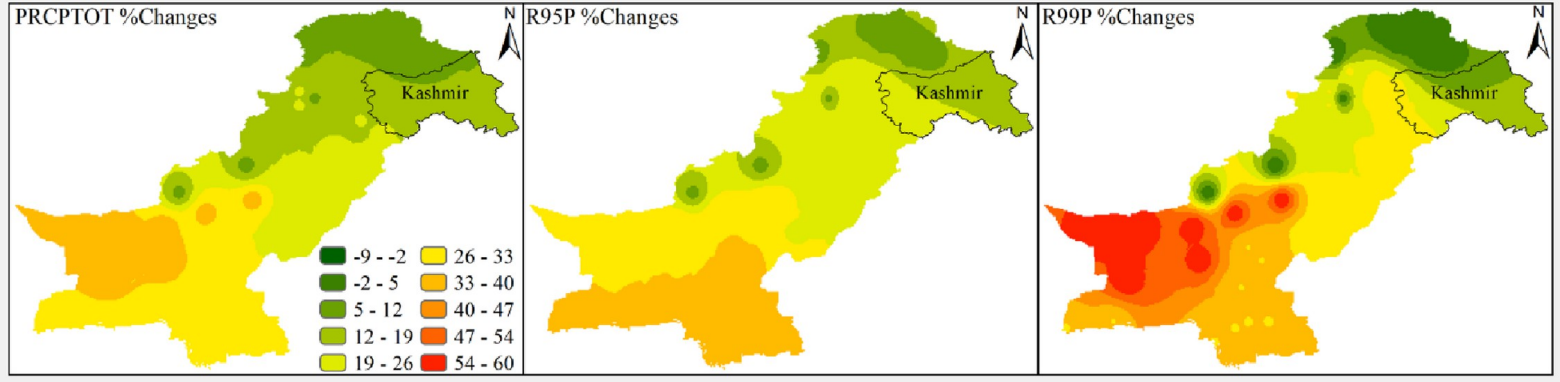
Spatial distribution of changes during 1991–2019 with respect to 1962–1990 of different climate extremes across Pakistan. The extremes are PRCPTOT, R95P, and R99P. The legend used in the first panel is applicable to all parts of this figure. The shapefile used for this figure is available at https://data.humdata.org/dataset/pakistan-union-council-boundaries-along-with-other-admin-boundaries-dataset.

Results related to climates’ extremes are given in Figs 3 and 4 for all zones. Most of the temperature extremes are significantly increased during 1991–2019. The number of frost days decreased significantly while growing season length and summer days increased significantly in zone 1 (Northern Pakistan). Interestingly, TN10P shows a significantly decreasing trend in 1991–2019 while increasing trend in 1962–1990. TX10P and TX90P have significantly decreasing and increasing trends, respectively, during 1991–2019. Most of the precipitation extremes have increasing trends during 1962–1990 and decreasing trends during 1991–2019. For example, PRCPTOT, R10mm, R20mm and R25mm have significantly increasing and significantly decreasing trend during 1962–1990 and 1991–2019, respectively. R95P decreased but R99P increased in zone 1. Spatial distribution of changes in selected temperatures’ extremes are presented in Figs 5 and 6 while changes in selected precipitations’ extremes are mentioned in Fig 7 for all climate zones.

**Fig 8:**
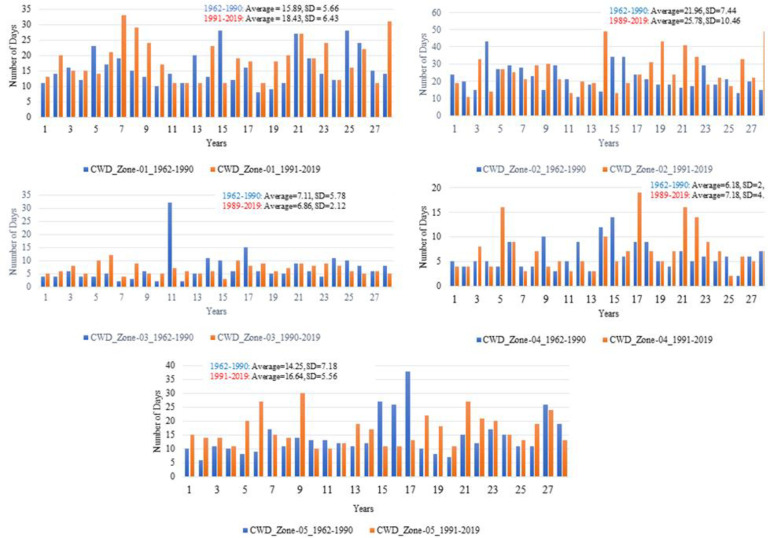
Number of consecutive wet days (CWD) for each climate zone and two independent durations. In each part of the figure, on x-axis and y-axis, years and number of days are mentioned.

**Fig 9:**
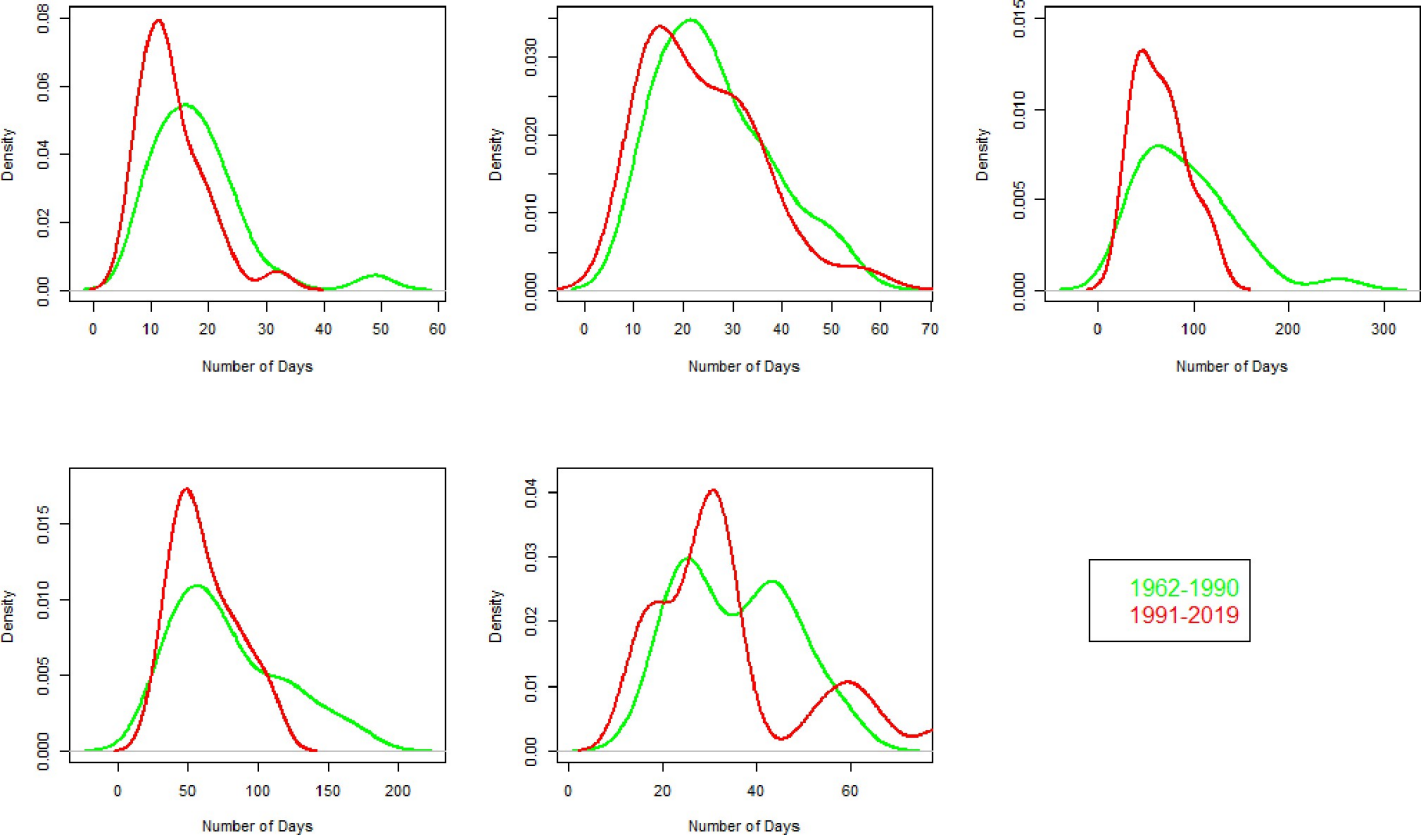
Probability density function of Consecutive dry days (CDD) for the duration of 1991–2019 and 1962–1990 in each climate zone. Green and red color represent 1962–1990 and 1991–2019, respectively. On x-axis the number of days and on y-axis the density of CDD is given.

**Fig 10:**
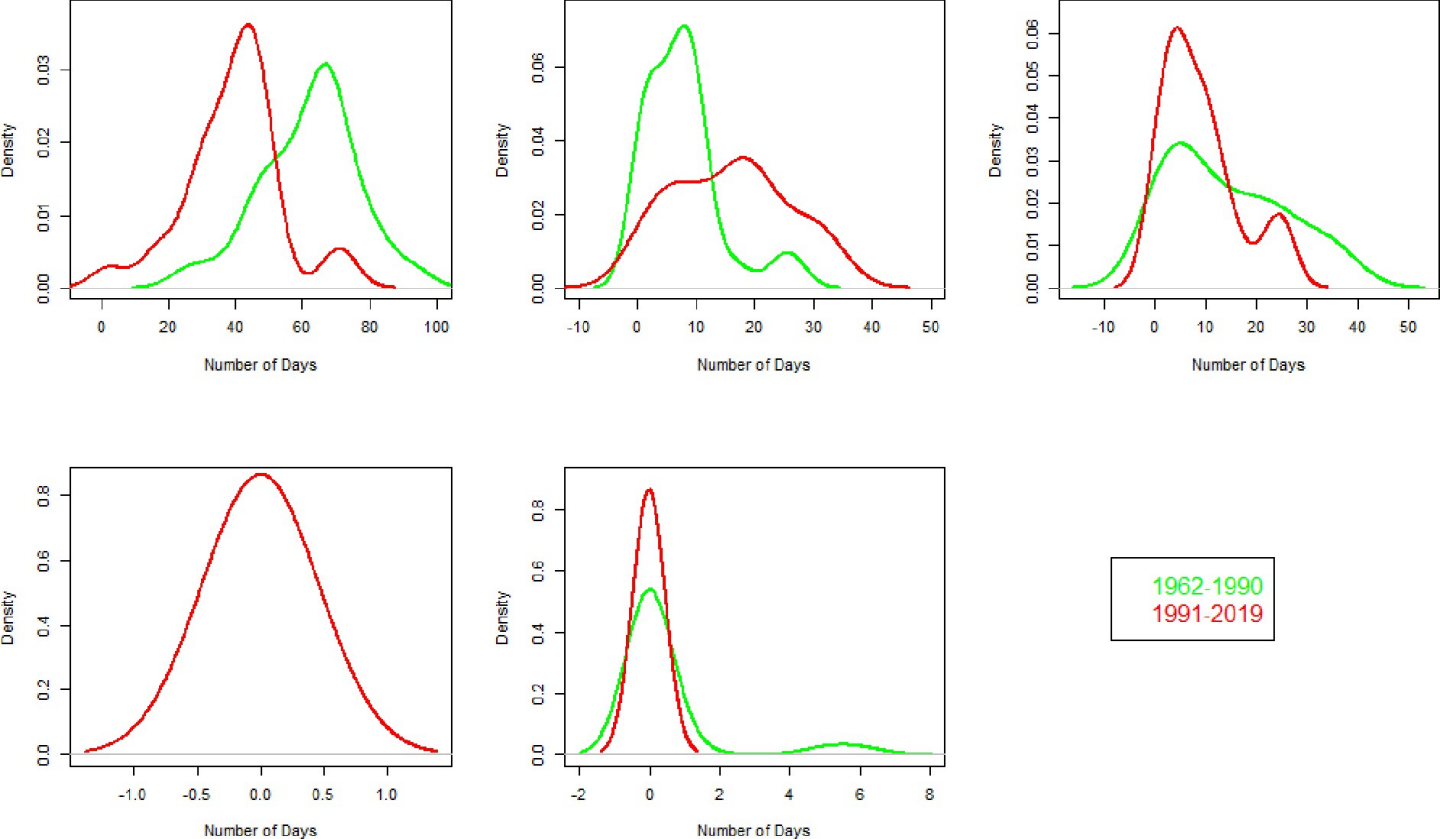
Probability density function of number of frost days (FD0) for the duration of 1991–2019 and 1962–1990 in each climate zone. Green and red color represent 1962–1990 and 1991–2019, respectively. On x-axis the number of days and on y-axis the density of FD0 is given.

The distribution of the number of CWD is displayed in Fig 8 for all zones. For zone 1, the average number of CWD increased from 16 to 19 in 1991–2019 compared to 1962–1990. A strong increase in variability is observed during 1991–2019. The maximum number of CDD is 60 and 40 during 1962–1990 and 1991–2019, respectively, given in Fig 9. The maximum number of Frost days (FD0) has decreased from 110 to 88 during 1991–2019 compared to 1962–1990 as shown in Fig 10.

#### Zone 2

The results of t-statistic (Table 2) show a significant increase in average temperature during MAM and JJA, increase during DJF, and insignificant decrease during SON. The mMK results for changes in maximum and minimum temperature indicate significantly increasing trend with values 0.0293 and 0.0938, respectively. On the other hand, average precipitation increased significantly throughout in all seasons. The mMK tests’ results (Table 3) show a significantly decreasing trend in precipitation with a magnitude of -0.0141.

Interestingly, this is the only region with the increasing number of frost days and decreasing GSL in both time slices. The SU25, TMAXMean, TN90p, TR20 and TX90p increased during the 1991–2019. The TMINMean increased significantly while TX10p and TN10p both decreased significantly during the 1991–2019. The CWD, WSDI, R10mm, R20mm and R25mm decreased during the 1991–2019. The CSDI, PRCPTOT, R95p and R99p decreased during the 1991–2019. The RX1day (Max 1-day precipitation amount) and Rx5day (Max 5-day precipitation amount) increased significantly and decreased during the 1962–1990 and 1991–2019, respectively.

The average number of CWD increased from 22 to 26 in the recent period. The standard deviation of CWD has increased from 8 (during 1962–1990) to 11 (during 1991–2019). The CDD (Fig 9) slightly reduced, with insignificant variability. The maximum number of FD0 recently increased from 34 to 47.

#### Zone 3

The average temperature significantly increased in this region, except a significant decreased for JJA with a value of -0.42. In contrast, the average precipitation increased significantly throughout except an insignificant increase during the DJF. The results of mMK test show decreasing trends in precipitation change and minimum temperature change while an increasing trend for maximum temperature change with the values -0.0890, -0.0637 and 0.0645, respectively.

Both GSL and SU25 increased in the recent period. TMAXMean has decreasing and significantly increasing trend in the first and second-time slice, respectively. The results of TMINMean are in contrast with TMAXMean. TN10P, TN90P, TNN, TNX, TR20 decreased in the recent time period. A significantly decreasing and increasing trend is noted in TX10P and TX90P in the second time slice, respectively. In addition, most of the precipitation extremes indicate an increasing trend during the first time slice except CDD, CSDI and WSDI with decreasing trend. In contrast, most of these extremes decreased except CDD, R10mm, R25mm and WSDI which have increasing trend during the 1991–2021. R95P, R99P and PRCPTOT have decreasing trend in the second time slice.

The average CWD and their standard deviation reduced from 8 and 6 during 1962–1990 to 6 and 3 during 1991–2019. The CDD (Fig 9) drastically decreased from 330 to 170 during 1962–1990 to 1991–2019 with decreased variability in the recent period. The average number of FD0 (Fig 10) reduced from 55 to 34 during 1962–1990 to 1991–2019.

#### Zone 4

The average precipitation and temperature changes in zone 4 are given in Table 2. The average temperature changed significantly with an increase during DJF, MAM, SON and decrease during JJA. The magnitude of change during DJF and JJA are highest with values 0.6908 and -0.1703, respectively. In addition, precipitation increased significantly only during JJA and SON. The mMK test shows a decreasing trend in precipitation and maximum temperature with values -0.0986 and -0.0212, respectively. The change in minimum temperature has significantly increasing trend with value a value of 0.158.

During the second time slice, GSL and SU25 increased and decreased, respectively. TMAXMean has an increasing trend in contrast to the significant increasing trend of TMINMean during 1991–2019. TN10P decreased during the second time slice compared to the first time slice. On the other hand, TN90P significantly increased during the second time slice. TX10P and TX90P show a decreasing and increasing trend, respectively, during the second time slice. During the second time slice, all precipitation extremes have decreasing trend except an increase in CWD.

The average number of CWD (Fig 8) has increased from 7 to 8 annually. The standard deviation of CWD increased from 3 to 5 during the 1991–2019. The maximum number of CWD is 19 during 1991–2019. The maximum number of CDD reduced from 225 to 149 during 1962–1990 and 1991–2019, respectively. No significant change with the average number of zero is observed in the distribution of FD0 (Fig 10).

#### Zone 5

The average temperature increased significantly except JJA with an insignificant increase. Changes in average precipitation, except MAM are highly significant. There is a significantly decreasing trend in precipitation and maximum temperature while significantly increasing trend for minimum temperature in this region. The values of mMK test statistic for change in precipitation, maximum, and minimum temperature are -0.0224, -0.0157 and 0.0560, respectively.

GSL, SU25, TMINMean, and TMAXMean have increasing trends during both time slices. TN10P has decreasing trend throughout while TN90P has decreasing and significantly increasing trends in the first and second time slice, respectively. TX10P and TX90P have decreasing trends during the second time period. Most of the precipitation extremes have increasing trends during both time slices. CDD, CSDI, R99P and WSDI have decreasing trends during the first time period while CSDI, PRCPTOT, R10mm, R95P and SDII have decreasing trends during the second time period.

The average number of CWD slightly increased from 15 to 17 annually during 1962–1990 to 1991–2019 (Fig 8). In contrast, the standard deviation of CWD decreased from 8 to 6 during 1962–1990 to 1991–2019. The distribution of CDD increased from 75 to 92 during 1962–1990 to 1991–2019. The number of FD0 reduced considerably from 8 to 2 during 1962–1990 to 1991–2019 (Fig 10).

### 4.2. Climate change and extremes’ analysis in HKH region (Present climatic status of Karakoram Anomaly)

The widely known Karakoram anomaly was investigated to see its present status by utilizing the updated observed meteorological data from the North of Pakistan as shown in Table 1. The stations are located between an altitude of 1,250 m a.s.l. at Chilas and 2,317 m a.s.l. at Skardu station. The data was analyzed seasonally to assess the contemporary climate change. The average temperature has an interesting pattern, specifically in winter (DJF), a significant increase at high altitude station (Skardu) of 0.97 and a significant decrease at the lowest station (Chilas) of –0.56 given in Table 4. Almost similar pattern is observed during MAM with the maximum increase at Astore and decrease at Chilas station. During JJA, average temperature decreased at all meteorological stations. Maximum and minimum decrease in the average temperature is noted at Gopis (with the magnitude of -1.42°C) and Darosh (with magnitude of -0.01°C), respectively. These changes are highly significant except at Chilas meteorological station. In SON, the average temperature has an increasing trend at all stations other than Bunji, Chilas and Gopis with decreasing trend.

**Table 4:** Test of significance of the changes between 1991–2019 and 1962–1990 about average temperature and precipitation for all seasons and meteorological stations situated in the Karakoram regions.

Variable	Season	Stations						
		Astore	Bunji	Chilas	Darosh	Gilgit	Gupis	Skardu
Difference of Average Temperature between 1991–2019 and 1962–1990	DJF	0.5082***	0.5404***	-0.5618***	0.7148***	0.8567***	-0.1942***	0.9677***
	MAM	0.6780***	0.5298***	-0.1238	0.6121***	0.7384***	-0.0823	0.7679***
	JJA	-0.2891***	-0.9248***	-0.1220**	-0.0137	-0.4890***	-1.4199***	-0.2753***
	SON	0.3906***	-0.3328***	-0.2666**	0.5326***	0.1510	-0.0157	0.0041
	**Overall**	0.3208**	**-0.0489**	-0.2671**	0.4600***	0.3121**	-0.4303***	0.3642**
Precipitation	DJF	0.2720***	0.1299***	0.1980***	0.1877**	-0.2388***	0.1456***	0.2984***
	MAM	-0.1176	-0.0347	0.0705	-0.3605***	-0.1836***	0.5083***	0.0513
	JJA	0.1502***	0.1719***	-0.2282**	0.03797	-0.3112**	0.3367***	0.0850***
	SON	-0.0786	0.0604***	0.0024	0.1001	-0.0727*	0.1046**	-0.3861**
	**Overall**	**0.0558**	0.0817**	**0.0098**	**-0.0099**	-0.2017**	0.2749***	**0.0119**

Note: Where “***”, “**” and “*” indicate significance of the test at 1%, 5% and 10% level of significance. DJF = December, January, February; MAM = March, April, May; JJA = June, July, August; SON = September, October, November.

Table 5 shows the seasonal trends in precipitation, maximum, and minimum temperature during 1962–1990. During DJF, precipitation has significantly increasing trend except Gopis where the trend is significantly decreasing. Maximum and minimum temperature results show significantly decreasing trend during JJA except Astore with an insignificant decrease in maximum temperature. These results are in line with Karakoram Anomaly during the 1962–1990. Table 6–8 show seasonal trend analysis for precipitation, maximum temperature and minimum temperature during 1991–1999, 2000–2009 and 2010–2019, respectively. Table 6 shows a comparison among three decades (1991–1999, 2000–2009, 2010–2019) using mMK test for precipitation. During 1991–1999, precipitation at Gilgit and Bunji increased while at Gopis and Skardu the trend decreased in DJF. On the contrary, the trend during 2000–2009 remained increasing. The trend changed again and decreased during 2010–2019 except Gopis with an insignificant increasing trend. The comparison of these three decades for maximum temperature is shown in Table 7. In JJA, the maximum temperature has a decreasing trend during 1990–1999 except Gilgit and during 2000–2009 at Gopis. The trend during 2010–2019 changed and increased in most of the stations except Gilgit with insignificant decreasing trend in JJA. Minimum temperature shows increasing trend in some parts (Bunji and Gilgit) and decreasing trend in others (Gopis and Skardu) during 1990–1999 and 2000–2009 as shown in Table 8. In contrast, during 2010–2019, minimum temperature shows increasing trend at these stations. These results show conflicting signals in climate. In the most recent decade of 2010–2019, precipitation decreased in DJF and temperature (maximum and minimum) increased in JJA. It is interesting to see a consistently decreasing trend in the SON in temperature (maximum and minimum) considering the overall thirty years period (1991–2019).

**Table 5:** Station and seasonal wise results of MK test and their statistical significance for precipitation, maximum and minimum temperature during the 1962–1990.

Station	DJF		MAM		JJA		SON	
	Kendall *τ*	P-Value	Kendall *τ*	P-Value	Kendall *τ*	P-Value	Kendall *τ*	P-Value
Precipitation (1962–1990)								
Astore	3.87e-02	0.011	1.63e-02	0.270			3.64e-02	0.020
Bunji	1.18e-02	0.461	5.91e-02	0.000	7.03e-02	6.82e-06	1.72e-02	0.280
Chilas	8.60e-02	5.44e-08	5.68e-02	0.000	8.49e-02	4.58e-08	5.79e-02	0.000
Darosh	1.54e-02	0.308	-5.37e-02	0.000	2.28e-02	0.136	-1.95e-02	0.204
Gilgit	4.34e-02	0.007	3.67e-02	0.017			7.05e-02	8.89e-06
Gopis	-6.32e-02	7.60e-05	-1.14e-01	1.95e-13	-8.49e-02	6.16e-08	-1.19e-01	8.97e-14
Skardu	5.05e-02	0.001	2.67e-02	0.083	9.11e-02	5.33e-09	2.02e-02	0.205
Maximum Temperature (1962–1990)								
Astore	-4.38e-02	0.001	3.42e-02	0.009	-6.06e-03	0.648	2.72e-03	0.838
Bunji	1.98e-02	0.140	2.75e-02	0.036	-8.89e-02	1.88e-11	-1.74e-02	0.188
Chilas	9.25e-03	0.490	3.76e-02	0.004	-3.13e-02	0.02	-6.33e-03	0.634
Darosh	-4.00e-02	0.003	6.11e-02	3.72e-06	-1.81e-02	0.173	-4.18e-02	0.002
Gilgit	6.74e-03	0.616	4.05e-02	0.002	-3.68e-02	0.005	-4.51e-03	0.734
Gopis	3.04e-02	0.023	5.77e-02	1.25e-05	-3.99e-02	0.002	-4.11e-02	0.002
Skardu	7.84e-02	4.73e-09	8.59e-02	7.90e-11	3.49e-02	0.008	3.49e-02	0.008
Minimum Temperature (1962–1990)								
Astore	1.23e-02	0.357	7.00e-02	1.28e-07	-2.93e-02	0.027	-3.48e-02	0.009
Bunji	-1.43e-01	2.2e-16	-5.93e-02	7.69e-06	-1.70e-01	2.2e-16	-1.57e-01	2.2e-16
Chilas	-5.26e-02	8.52e-05	4.24e-02	0.001	-4.98e-02	0.000	-4.22e-02	0.001
Darosh	-1.06e-01	2.68e-15	-3.04e-03	0.818	-1.11e-01	2.2e-16	-8.56e-02	1.20e-10
Gilgit			5.03e-03	0.704	-3.74e-02	0.005	-4.54e-02	0.000
Gopis	1.68e-02	0.21	5.08e-02	0.000	-6.19e-02	3.08e-06	-8.87e-02	2.53e-11
Skardu	6.14e-02	4.46e-06	-6.95e-03	0.600	-7.90e-02	2.78e-09	-5.60e-02	2.45e-05

**Table 6:** Seasonal MK test results in the Karakoram region for precipitation. The table shows the Kendall *τ* and their significance for the 1990–1999, 2000–2009 and 2010–2019.

Station	DJF		MAM		JJA		SON	
Station	Kendall *τ*	P-Value	Kendall *τ*	P-Value	Kendall *τ*	P-Value	Kendall *τ*	P-Value
Precipitation (1990–1999)								
Astore	-6.75e-02	0.007	-1.06e-02	0.663	-2.46e-02	0.327	-2.40e-02	0.357
Bunji	4.34e-02	0.104	8.94e-02	0.000	4.04e-02	0.121	4.50e-02	0.091
Chilas	4.21e-02	0.114	4.48e-03	0.862	1.66e-02	0.524	1.16e-03	0.965
Darosh	-9.16e-0	0.000	-3.57e-02	0.140	2.14e-03	0.934	9.95e-03	0.702
Gilgit	2.46e-02	0.351	2.67e-02	0.289	6.03e-02	0.018	-1.62e-02	0.539
Gopis	-1.62e-03	0.951	4.77e-02	0.069	6.50e-02	0.014	-6.71e-02	0.012
Skardu	-3.37e-02	0.190	3.81e-02	0.133	7.15e-02	0.006	6.41e-02	0.016
Precipitation (2000–2009)								
Astore	-5.21e-02	0.044	-1.02e-01	5.227e-05	-4.62e-02	0.068	-3.80e-02	0.146
Bunji	3.58e-02	0.180	-1.20e-02	0.645	2.65e-02	0.307	-3.15e-02	0.236
Chilas	1.22e-01	4.14e-06	4.03e-02	0.118	1.13e-01	1.52e-05	5.52e-02	0.038
Darosh	6.47e-02	0.011	2.31e-02	0.355	-5.41e-02	0.038	-3.77e-02	0.147
Gilgit	6.84e-02	0.009	1.31e-02	0.608	-3.86e-02	0.127	-2.61e-03	0.921
Gopis	5.69e-02	0.033	-3.12e-02	0.234	-8.10e-02	0.002	-6.50e-02	0.015
Skardu	5.38e-02	0.037	-1.41e-02	0.584	-2.92e-02	0.254	8.34e-04	0.975
Precipitation (2010–2019)								
Astore	-1.30e-01	7.53e-06	1.34e-03	0.961	-6.15e-03	0.828	-3.59e-02	0.219
Bunji	-3.37e-03	0.910	-7.70e-03	0.787	-1.60e-02	0.574	-2.59e-02	0.379
Chilas	1.57e-01	9.5e-08	-3.05e-02	0.286	-1.18e-02	0.683	-9.35e-02	0.001
Darosh	6.88e-03	0.812	-1.27e-02	0.646	-1.95e-02	0.502	-4.89e-02	0.095
Gilgit	-1.03e-02	0.728	-3.62e-03	0.898	-2.35e-02	0.401	-6.59e-02	0.023
Gopis	4.52e-03	0.881	7.16e-02	0.014	6.73e-03	0.818	-7.48e-03	0.802
Skardu	-1.54e-01	1.98e-07	1.26e-02	0.66	4.69e-02	0.102	-2.52e-02	0.395

**Table 7:** Seasonal MK test results in the Karakoram region for maximum temperature. The table shows the Kendall *τ* and their significance for the 1990–1999, 2000–2009 and 2010–2019.

Station	DJF		MAM		JJA		SON	
Station	Kendall *τ*	P-Value	Kendall *τ*	P-Value	Kendall *τ*	P-Value	Kendall *τ*	P-Value
Maximum Temperature (1990–1999)								
Astore	5.18e-02	0.021	9.51e-02	1.68e-05	9.22e-02	3.24e-05	-3.05e-02	0.169
Bunji	8.52e-02	0.000	6.23e-02	0.005	-1.41e-02	0.525	-4.92e-02	0.027
Chilas	1.32e-01	3.35e-09	7.49e-02	0.001	9.76e-03	0.659	-2.66e-02	0.232
Darosh	2.28e-01	2.2e-16	8.28e-02	0.000	-6.26e-02	0.005	-1.44e-02	0.515
Gilgit	1.40e-01	3.23e-10	8.06e-02	0.000	3.32e-03	0.881	-2.45e-02	0.27
Gopis	1.81e-01	1.90e-15	7.74e-02	0.000	-1.13e-01	4.14e-07	-5.00e-02	0.025
Skardu	3.85e-02	0.084	7.03e-02	0.001	-7.74e-02	0.000	-7.57e-02	0.001
Maximum Temperature (2000–2009)								
Astore	-5.60e-02	0.012	9.88e-02	8.00e-06	6.90e-02	0.002	-6.35e-02	0.004
Bunji	-8.24e-02	0.000	3.46e-02	0.119	-3.17e-02	0.156	-8.61e-02	0.000
Chilas	-1.08e-01	1.53e-06	4.33e-02	0.050	-7.52e-03	0.735	-6.58e-02	0.003
Darosh	-9.40e-02	2.54e-05	-1.08e-02	0.626	-3.63e-02	0.101	-8.58e-02	0.000
Gilgit	-1.15e-01	3.1e-07	3.03e-02	0.171	-2.59e-02	0.244	-1.06e-01	2.01e-06
Gopis	-1.17e-01	2.08e-07	5.18e-02	0.019	2.27e-03	0.919	-7.33e-02	0.001
Skardu	-1.13e-02	0.614	3.27e-02	0.139	-1.47e-02	0.508	-1.18e-01	1.11e-07
Maximum Temperature (2010–2019)								
Astore	9.78e-02	0.000	9.92e-02	6.24e-05	9.77e-02	8.57e-05	-3.85e-02	0.122
Bunji	1.33e-01	1.54e-07	6.51e-02	0.008	1.90e-02	0.446	-8.71e-02	0.000
Chilas	1.01e-01	6.28e-05	8.18e-02	0.001	1.14e-01	4.30e-06	-7.29e-02	0.003
Darosh	1.00e-01	5.67e-05	8.03e-02	0.001	6.04e-02	0.014	-5.94e-02	0.016
Gilgit	3.87e-02	0.127	5.08e-02	0.041	-1.30e-02	0.602	-9.77e-02	9.50e-05
Gopis	9.21e-02	0.000	5.88e-02	0.018	1.17e-02	0.640	-8.01e-02	0.001
Skardu	7.72e-02	0.002	8.19e-02	0.001	1.79e-02	0.4704	-1.50e-01	1.58e-09

**Table 8:** Seasonal MK test results in the Karakoram region for minimum temperature. The table shows the Kendall *τ* and their significance for the 1990–1999, 2000–2009 and 2010–2019.

Station	DJF		MAM		JJA		SON	
Station	Kendall *τ*	P-Value	Kendall *τ*	P-Value	Kendall *τ*	P-Value	Kendall *τ*	P-Value
Minimum Temperature (1990–1999)								
Astore	3.60e-02	0.11	1.51e-01	8.73e-12	1.61e-01	4.38e-13	-3.14e-04	0.989
Bunji	-4.88e-02	0.030	7.85e-02	0.000	1.10e-03	0.960	-4.94e-02	0.026
Chilas			1.12e-01	5.79e-07	1.41e-01	3.23e-10	-7.65e-02	0.001
Darosh	1.13e-01	4.54e-07	1.03e-01	3.33e-06	6.64e-03	0.764	-5.04e-02	0.023
Gilgit	-1.56e-01	2.88e-12	9.95e-02	7.01e-06	4.19e-02	0.058	-3.51e-02	0.114
Gopis	3.91e-02	0.086	1.05e-01	2.72e-06	-3.01e-02	0.178	-2.01e-02	0.368
Skardu	-6.44e-02	0.004	9.08e-02	4.04e-05	-1.24e-02	0.575	-3.72e-02	0.094
Minimum Temperature (2000–2009)								
Astore	2.76e-03	0.902	1.07e-01	1.56e-06	7.35e-02	0.001	-7.00e-02	0.001
Bunji	7.40e-02	0.001	7.49e-02	0.001	1.13e-01	3.77e-07	5.98e-02	0.007
Chilas	1.59e-01	3.66e-12	8.46e-02	0.000	3.84e-02	0.089	-3.69e-02	0.100
Darosh	-1.91e-01	2.2e-16	-5.36e-02	0.015	-2.88e-01	2.2e-16	-2.30e-01	2.2e-16
Gilgit	1.31e-01	5.39e-09	1.24e-01	2.61e-08	1.84e-01	2.2e-16	3.98e-02	0.074
Gopis	-1.94e-01	2.2e-16	2.56e-02	0.250	-6.04e-02	0.007	-1.43e-01	1.46e-10
Skardu	1.05e-02	0.635	-3.29e-03	0.882	-1.48e-01	2.18e-11	-1.51e-01	9.57e-12
Minimum Temperature (2010–2019)								
Astore	-9.41e-03	0.709	5.45e-02	0.028	1.23e-01	9.29e-07	-1.08e-01	1.55e-05
Bunji	2.65e-01	< 2.2e-16	1.41e-01	1.30e-08	1.62e-01	9.06e-11	-7.09e-03	0.777
Chilas	1.18e-01	2.90e-06	9.01e-03	0.716	-1.40e-01	1.66e-08	-1.11e-01	8.16e-06
Darosh	1.31e-03	0.958	-5.57e-03	0.822	1.57e-02	0.527	-7.7e-02	0.001
Gilgit	8.88e-02	0.000	4.42e-02	0.078	1.23e-01	1.16e-06	-9.07e-02	0.000
Gopis	2.13e-01	< 2.2e-16	6.32e-02	0.012	2.14e-02	0.397	-1.11e-01	1.05e-05
Skardu	1.08e-01	1.70e-05	4.59e-02	0.063	2.01e-02	0.418	-4.81e-02	0.053

**Fig 11:**
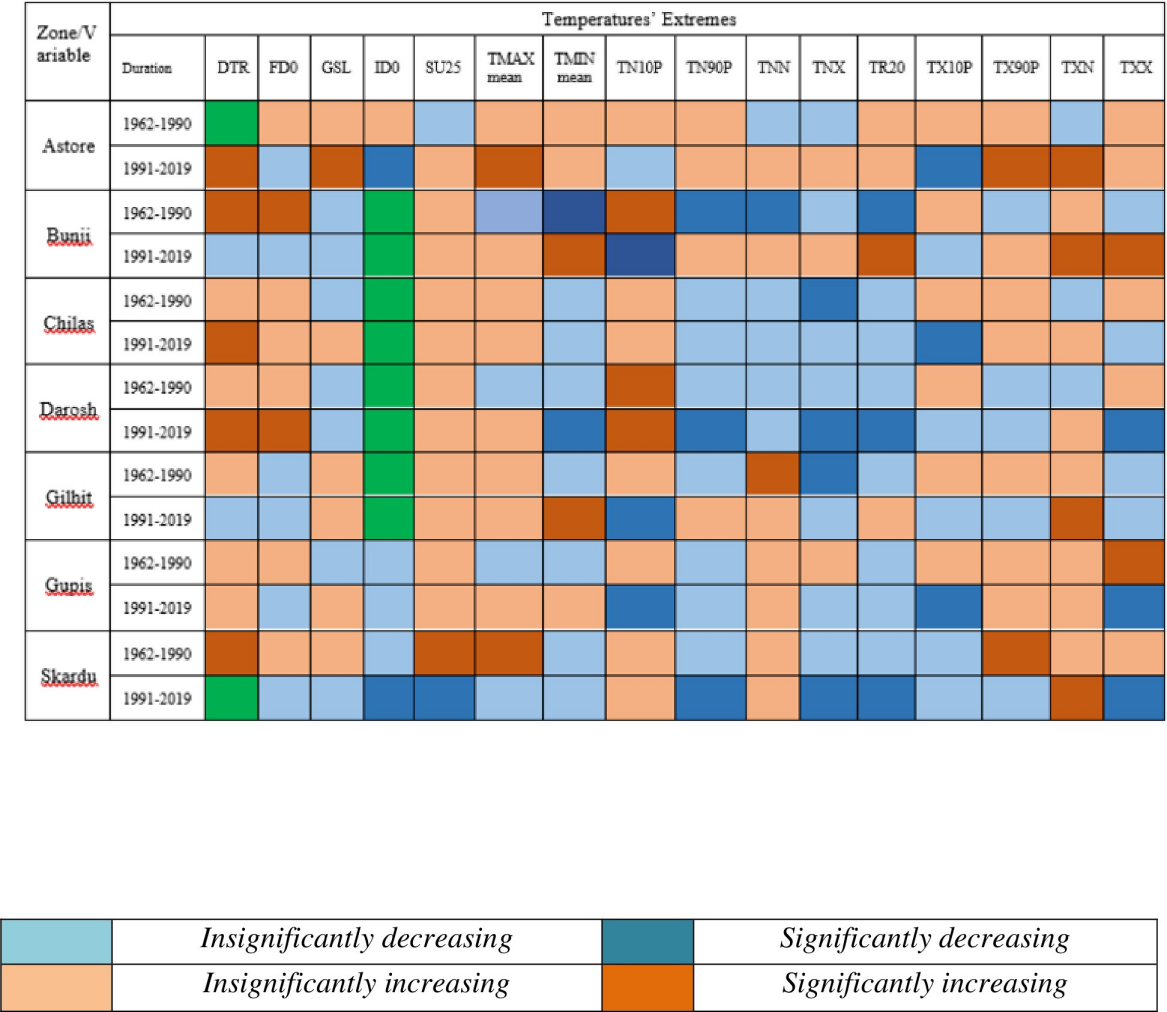
Station-wise climate extremes related to temperature for each climatic zone and two independent time duration in Karakoram region. Each color has specific meaning and given with Fig 12. *Note*: *The colors represent different status for each extreme mentioned in these figures*. *Green color shows no changes*.

The station-wise climate extremes in the northern Pakistan are shown in Fig 11. The results show that SU25 increased throughout except Skardu during 1991–2019. TN10P (cold nights) decreased significantly at some of the stations located in the Northern Pakistan during 1991–2019. TN90P (warm nights), TX10P (cold days) TR20 (tropical nights) have decreasing trend during 1991–2019 in most parts of this region. TX90P (warm days) has mixed trend, decreasing at Darosh, Gilgit and Skardu and increasing trend at the remaining stations.

**Fig 12:**
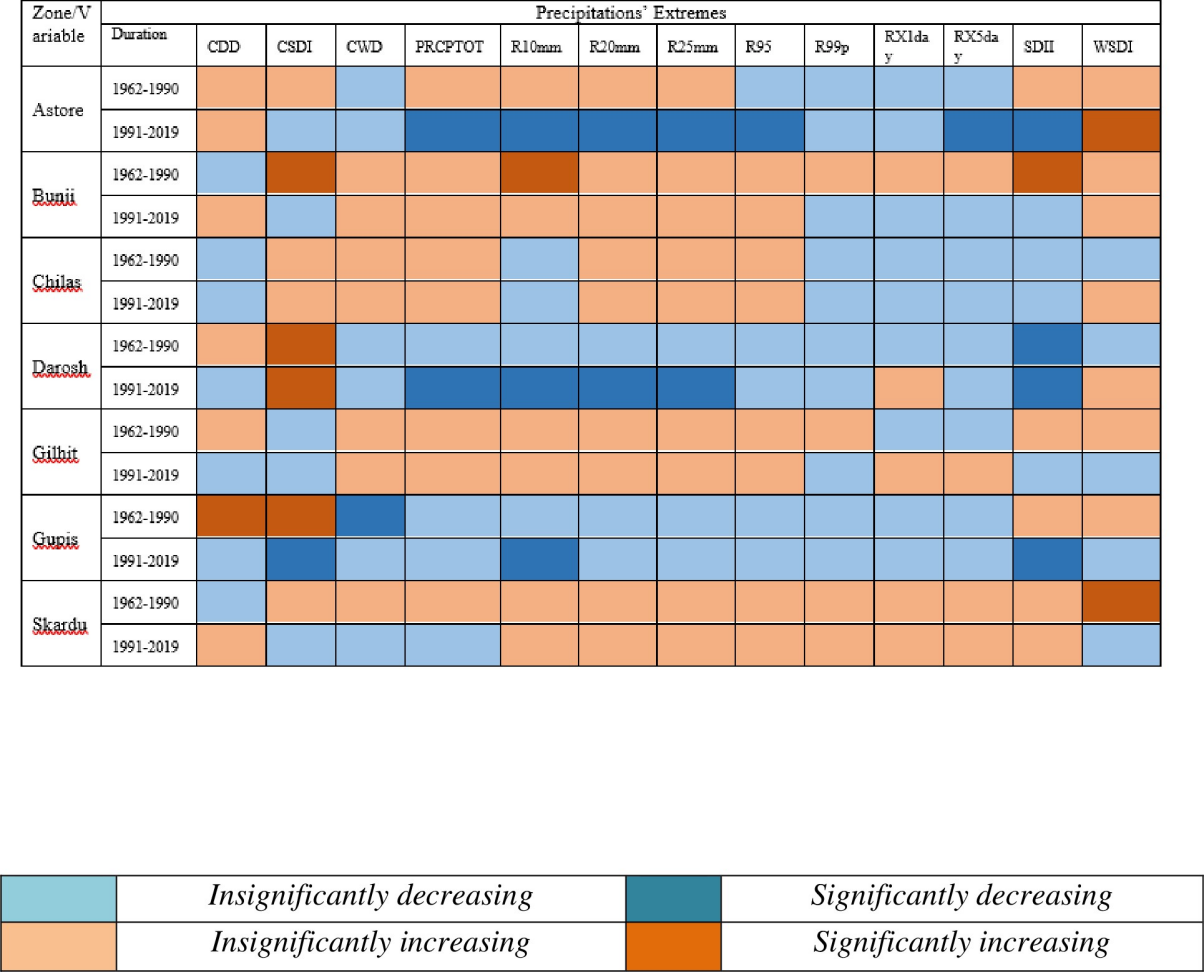
Station-wise climate extremes related to precipitation for each climatic zone and two independent time duration In Karakoram region. Each color has specific meaning and given below. *Note*: *The colors represent different status for each extreme mentioned in these figures*. *Green color shows no changes*.

Fig 12 shows decreasing trend in precipitation extremes except CDD and WSDI in western and eastern parts. However, CWD, R10mm, R20mm, R25mm, R95 have increasing trends while CWD and PRECPTOT show decreasing trends in the eastern-central Karakoram.

## 5. Discussion

Zone 1 is rich with water resources (snow and glaciers). Therefore, the changes in climate and climate extremes may have significant impacts on water availability in dry summers, the frequency of flooding, and Glacier Lake Outburst Floods (GLOFs) in the future. Zone 2 comprise Monsoon dominated region receiving more than 60% precipitation during Monsoon season [77–79]. The seasonal changes in precipitation and temperature may affect the demand and supply of water and ultimately the crop’s production. In addition, Zone 3 is the south-west part of Pakistan receiving low amount of rainfall round the year. Precipitation extremes may contribute less to cause flooding in the region. Zone 4 faces shortage of rainfall and have severe rainfall decrease, which can further exacerbate the shortage of water for drinking, agriculture and other purposes. Zone 5 is mainly comprised of Punjab province dominating the agriculture production of the country. According to our results, R99P has increasing trend in this region with the likelihood of intense rainfall and may cause flash flooding in the future.

As UIB is melt water dominated, therefore, the latest information of the cryosphere is important. In addition, changes in climate variables can significantly affect downstream water’s availability for millions of inhabitants [44,80]. Most of the HKHs’ glaciers are retreating since the mid-19^th^ century [41,42,81]. In addition, some recent studies found that mass balance of the glaciers in the Karakoram are nearly in balance during 1971–2010 [41,82,83]. Muhammad et al. [76] derived the mass balance of glaciers in the sub-basins of UIB with negative mass balance except Shigar and Hunza in the central and western Karakoram, respectively. In another study, Muhammad et al. [84] investigated the mass balance of glaciers in Astore (a sub basin of UIB) and concluded that the glaciers in the basin (western Himalaya) are nearly in balance during 2000–2016 similar to the neighboring glaciers in the adjacent Karakoram. In addition to the direct glacier observations, climate data also show interesting signals and indirectly indicate glacier’s health status. For instance, [44] noted that mean temperature decreased at the stations (Bunji, Gilgit and Skardu) lying in the Karakoram region during 1961–2000. Their analysis shows that a decrease of 1°C in mean summer temperature can cause a reduction of ~20% in summer runoff. Fowler and Archer [44] concluded an expansion in contrast to the widespread retreat in the remaining parts of the HKH region. Latif et al. [85] investigated trends in maximum, minimum temperature in the northern part of Pakistan. They observed that maximum temperature has mixed (upward and downward) trend while the cooling in minimum temperature become stronger in the recent past. Ali et al. [86] investigated temperatures’ changes in the UIB by considering meteorological data for two stations. They noted that the mean temperature increased by 0.63°C in Skardu while decreased by − 0.137°C in Gilgit in the recent past. Shahzaman et al. [87] concluded in their study that drought has been observed over central and northern Pakistan during 1982–2019 by integrating various data types. Proportion of South Asian population exposer to heatwaves is increasing under the shared socioeconomic scenarios in the twenty first century, however, the northern areas of Pakistan are unaffected presently [88].

The mass balance of the glacier is heavily dependent on the winter accumulation and summer ablation strongly influenced by precipitation and temperature among other factors [89]. This study provides up to date changes in precipitation and temperature in some parts in the northern Pakistan (4 meteorological stations are in the Karakoram region). We found significant increase in winter average precipitation and decrease in summer average temperature particularly at the meteorological stations in the Karakoram region during the 1962–1990 (Table 1). However, the mMK test indicates a decreasing trend (during DJF) in precipitation and increasing trend (during JJA) in temperature in the recent period of 2010–2019 in the Karakoram which is not aligned with the Karakoram Anomaly. Our findings are an indication of glaciers mass loss in future in the Karakoram region. These stations are situated in the valley below the glacier terminating zones, however, an indirect clue can be inferred based on climate change and extreme events regarding future trends. Our results suggest further investigation of glaciers mass balance in the region in future for better understanding of glaciers’ health and associated impacts. The downstream water availability in UIB is dominated by snow and glacier melt water particularly in summer, therefore, the climate change assessment can indicate indirect signals for water availability. In addition, climate extremes can provide guidelines to policy makers and relevant stakeholder for future climate trends and meltwater resources in the country.

## 6. Conclusions

During DJF and MAM, average temperature increased in all climate zones. A decreasing trend during JJA in zone 1, 3, 4 and during SON in zones 1 and 2 were noted in the average temperature. Precipitation indicated mixed trend in different zones. Mann-Kendal test indicated trend in maximum and minimum temperature except zone-4 where the maximum temperature has no trend. On the average, CWDs increased in all zones except in zone 3 where it decreased. The number of SU25 increased in all climate zone except zone 4.

In the Karakoram region, temperature decreased significantly during JJA. Also, precipitation increased significantly in this region except a significant decrease in Gilgit during DJF. MK test suggested increasing trend in precipitation and decreasing trend in maximum and minimum temperature during 1962–1990 and closely in line with Karakoram Anomaly. However, precipitation (in DJF) and temperature (in JJA) have decreasing and increasing trend, respectively, during the most recent period (2010–2019), which do not support the notion of Karakoram Anomaly. These findings suggested that the glacier may lose mass in future rather than gaining or stable conditions. Climate extremes have mixed trends over North Pakistan. For instance, summer days increased, and cold nights have mixed trend. Warm nights decreased except in western Karakoram.

The key takeaways are:

1. A change is noted in the Climate (maximum, minimum temperature and precipitation) across the country during the analysis period.
2. TN10p, TX10p and TR20, CSDI and PRECPTOT have decreasing trend particularly during the 1991–2019 in most parts of the country.
3. Precipitation has increasing trend in DJF while temperature has decreasing trend in JJA during the 1962–1990 at the stations located in the Karakoram region.
4. During the last decade (2010–2019), precipitation and temperature have decreasing and increasing trend, respectively, at the stations located in the Karakoram region.
5. Based on the points in 3 and 4, the glaciers in the Karakoram region may loses mass balance slightly in the near future, which is in contrast to the famous Karakoram Anomaly.

## Supporting information

S1 Data (XLSX)
